# Prevalence, Species Diversity, and Risk Factors of Tick Infestation in Cattle From District Peshawar, Pakistan

**DOI:** 10.1002/vms3.70642

**Published:** 2025-10-09

**Authors:** Murad Ali Khan, Zohaib Ali, Abdur Rehman, Raheela Murad, Shabana Naz, Rifat Ullah Khan, Solomon Tesfay, Marco Ragni, Ibrahim A. Alhidary

**Affiliations:** ^1^ College of Veterinary Sciences Faculty of Animal Husbandry and Veterinary Sciences The University of Agriculture Peshawar Pakistan; ^2^ Department of Zoology Government College University Faisalabad Pakistan; ^3^ Department of Biology CNCS Mekelle University Mekelle Ethiopia; ^4^ Department of Soil Plant and Food University of Bari Aldomoro Italy; ^5^ Department of Animal Production College of Agriculture and Food Sciences King Saud University Riyadh Saudi Arabia

**Keywords:** cattle breeds, epidemiological analysis, Theileria annulata, tick infestation, vector‐borne diseases

## Abstract

This study investigated the prevalence, diversity, and risk factors of tick infestation and *Theileria annulata* infection in cattle from District Peshawar, Pakistan. A total of 322 cattle of different breeds, ages, and sexes were examined between January and March 2024. The overall prevalence of tick infestation was 35.4%, with females more frequently infested than males. Exotic and crossbred cattle, particularly Holstein Friesian and Jersey, along with calves, showed higher susceptibility compared to indigenous breeds and older cattle. Morphological identification revealed *Rhipicephalus microplus* as the most prevalent species, followed by *Hyalomma anatolicum anatolicum*, while other species occurred at lower frequencies. Species distribution varied significantly across breeds, with *R. microplus* predominating in Sahiwal cattle and *H. anatolicum* strongly associated with Holstein Friesian. Molecular screening confirmed *T. annulata* infection, with the highest prevalence detected in female *H. anatolicum*, whereas *R. microplus* showed relatively low infection rates. Ecological analysis indicated moderate species diversity overall, with Holstein Friesian cattle exhibiting the greatest richness and dissimilarity in tick fauna compared to indigenous breeds. Tick burden severity was mostly moderate, and co‐infestation patterns were dominated by combinations of *H. anatolicum* and *R. microplus*. This study is the first to integrate molecular detection of *T. annulata* with ecological indices of tick diversity in cattle of Peshawar, providing a comprehensive picture of vector–host–pathogen interactions. The identification of breed‐ and age‐specific vulnerabilities highlights the need for targeted tick control strategies. These findings have direct implications for designing sustainable management programs aimed at reducing the economic and health burden of tropical theileriosis in endemic regions.

## Graphical abstract

This study documents the prevalence and risk factors of cattle tick infestation in District Peshawar using morphological and molecular identification. Ecological diversity indices reveal moderate tick diversity, while risk factor analysis highlights breed, age, and sex as significant predictors of infestation.

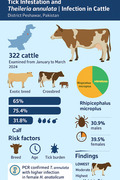

## 1 Introduction

Ticks are among the most significant ectoparasites affecting livestock worldwide, both in terms of direct damage and as vectors for various pathogens, notably protozoa, bacteria, and viruses (S. Ali et al. 2024; Zheng et al. 2024). In cattle, ticks transmit a wide range of diseases that impair productivity, compromise animal health, and impose economic burdens on livestock owners, particularly in tropical and subtropical regions (Strydom et al. 2023; Al‐Hoshani et al. 2023). Of these diseases, tropical theileriosis, caused by the protozoan parasite *Theileria annulata*, is among the most important tick‐borne diseases (TBDs) in bovines in Asia, the Middle East, and North Africa (de la Fuente et al. 2023).

*T. annulata* is transmitted primarily by ticks of the *Hyalomma* genus, especially *Hyalomma anatolicum anatolicum*, which is widely distributed across the Indian subcontinent, including Pakistan (S. Ali et al. 2024). Infection with *T. annulata* results in lymphoproliferative disease characterized by high fever, anemia, lymph node enlargement, respiratory distress, and often death in susceptible animals if untreated (de la Fuente et al. 2023). The disease is particularly severe in exotic breeds such as Holstein Friesian and Jersey, which are less adapted to local vector pressure and climate, making it a critical constraint to cross‐breeding programs in endemic areas (Tharwat et al. 2024; Bashiru, and Oseni 2025).

Cattle are a major component of this sector, especially in rural areas such as the Peshawar district of Khyber Pakhtunkhwa, where they are essential for milk, meat, draught power, and livelihood. Despite this importance, comprehensive epidemiological data on tick infestation and TBD prevalence in these regions remain sparse, particularly regarding the diversity of tick species and their role in pathogen transmission. This data gap hinders the formulation of targeted and region‐specific control strategies (Aslam et al. 2023).

Understanding the ecology and species composition of ticks infesting cattle is essential for designing effective tick management programs. Various studies in Pakistan have documented the presence of multiple tick genera, including *Hyalomma*, *Rhipicephalus*, *Haemaphysalis*, and *Boophilus*, each differing in vector potential, seasonality, and host preference (Rooman et al. 2021; Hussain et al. 2024). Moreover, the prevalence and intensity of tick infestation are influenced by several host‐associated factors such as age, sex, breed, and management practices, as well as environmental variables like temperature and humidity (Aslam et al. 2023). Crossbred and exotic cattle are frequently reported to harbor heavier tick burdens compared to indigenous breeds, largely due to differences in skin texture, grooming behavior, and immune response (Teel and Hairgrove 2024).

Advancements in molecular diagnostic techniques, especially polymerase chain reaction (PCR), have significantly improved the detection and characterization of *T. annulata*. PCR targeting the 18S ribosomal RNA gene is widely used due to its high sensitivity, specificity, and ability to differentiate closely related *Theileria* species (Kumar et al. 2022; Babiker et al. 2024). Molecular characterization not only confirms infection status but also facilitates phylogenetic analysis, allowing researchers to understand the genetic diversity and evolutionary relationships among field isolates. This information is critical in the context of emerging pathogen variants, treatment, and regional disease surveillance (Eltaly et al. 2023; Tumer et al. 2023).

Despite the availability of molecular tools, most rural and tribal districts in Pakistan remain under‐investigated in terms of tick ecology and tick‐borne pathogens. Peshawar, located near the Pakistan–Afghanistan border, is one such underserved district where cattle rearing is practiced under traditional, low‐input systems (Z. Iqbal et al. 2022). In these settings, communal grazing, minimal tick control practices, and weak veterinary infrastructure contribute to high exposure of animals to ticks and associated pathogens (Jamil et al. 2023). Ticks are not only major vectors of *T. annulata* but also exert significant economic losses through reduced productivity, increased treatment costs, and animal mortality, which are especially burdensome for resource‐limited farmers (Strydom et al. 2023; de la Fuente et al. 2023). Moreover, climate variability is predicted to further intensify tick distribution and disease transmission risks in low‐ and middle‐income countries (Bashiru and Oseni 2025).

Without baseline data on tick species diversity, infestation rates, and *T. annulata* prevalence in districts like Peshawar, it is difficult to design effective interventions or implement region‐specific control strategies. Previous studies have highlighted the clinical and economic consequences of tropical theileriosis in livestock, including alterations in hematobiochemical profiles, organ dysfunction, and mortality (Tharwat et al. 2024; Tumer et al. 2023; Aslam et al. 2023). In addition, co‐infestation by multiple tick species, each with different vector competencies, may modify transmission dynamics, exacerbate disease severity, and increase the likelihood of multiple pathogen co‐transmission (Rocha et al. 2022; Zheng et al. 2024). These factors complicate control efforts and highlight the need for detailed epidemiological studies in under‐researched tribal districts.

Therefore, documenting tick species composition, co‐infestation patterns, and identifying the dominant vectors of *T. annulata* in Peshawar is an urgent necessity. The current study addresses this gap by integrating field surveillance with molecular diagnostics to investigate the epidemiology of tick infestation and *T. annulata* infection in cattle from Peshawar. Such approaches are essential for evidence‐based, region‐specific tick management programs and for reducing the broader economic impact of tropical theileriosis on rural livelihoods (Al‐Hoshani et al. 2023).

## 2 Materials and Methods

### 2.1 Study Area and Geographic Location

The present study was conducted in District Peshawar, located in Khyber Pakhtunkhwa, northwest Pakistan. The district lies between latitude 34.0150°N and longitude 31.5249°E. It is bordered by Charsadda to the north, Nowshera to the east, Khyber Pass to the west, and Khyber district to the south. The total geographical area of the district is approximately 1,257 km^2^, with a human population of around 2.3 million. The cattle population in Peshawar is estimated at 223,150 heads as per the Pakistan Economic Survey 2023–24. The study was carried out over a period of three months, from January to March 2024, coinciding with the winter season in the region. During this period, average environmental conditions (temperature 6°C–18°C, relative humidity 45%–65%) were recorded from the Pakistan Meteorological Department to account for climatic factors influencing tick activity.

### 2.2 Animal Selection Criteria

Cattle of varying breeds (Holstein Friesian, Jersey, Sahiwal, and Crossbred), ages (calves, sub‐adults, and adults), and sexes (male and female) were selected for tick examination. Animals were randomly chosen from different localities and management systems within the district to ensure representation and minimize sampling bias. Randomization was performed by visiting farms sequentially and selecting animals using systematic random sampling (every third animal encountered), thus avoiding overrepresentation of specific herds.

### 2.3 Sample Size

According to the formula given by Thrusfield (2018), the sample size was 322.

$$n=\frac{Z^{2}\times p\times \left(1-p\right)}{E^{2}}$$

where *n* is the required sample size, *Z* is the *Z*‐score (standard score corresponding to the desired confidence level), *p* is the estimated prevalence of the condition in the population (expressed as a decimal), and *E* is the margin of error (precession expressed as decimal).

$$n=\frac{1.96^{2}\times 0.3\times \left(1-0.3\right)}{0.05^{2}}=322$$

This calculation yielded a minimum of 322 animals, which were included in the study to ensure statistical validity and reliable prevalence estimates.

### 2.4 Tick Collection and Preservation

A total of 322 cattle were examined, yielding 2846 ticks (mean burden: 8.8 ticks/animal). This sample size of ticks was not predetermined but was a direct outcome of the cattle examination, and it was considered sufficient for robust prevalence estimation and species‐level diversity analysis. Ticks were manually collected from all regions of each sampled animal using fine‐tipped forceps to avoid selection bias toward known predilection sites. The animal's body was thoroughly examined for tick presence. Each collected specimen was stored in a separate labelled vial indicating the host ID, breed, sex, collection site, and date. To prevent degradation, ticks were cleaned of any dermal debris and immersed in 70% ethanol immediately after collection. Care was taken to avoid damage to the tick's body, especially the mouthparts, to preserve morphological features for accurate identification. All instruments (forceps, sample containers, and microscopes) were sterilized and calibrated before use. The stereomicroscope (Nikon SMZ‐745T) was calibrated at 10× magnification daily using a stage micrometer to ensure measurement accuracy during morphological identification.

### 2.5 Morphological Identification of Ticks

Tick identification was carried out at the Parasitology Laboratory, College of Veterinary Sciences, using a stereomicroscope (40× magnification). Morphological features such as capitulum structure, scutum shape, and festoons were examined. Identification keys and standard taxonomic references by Hoogstraal (1956), J. B. Walker et al. (2000), A. R. Walker (2003), and Nava et al. (2018) were used to confirm species‐level classification.

### 2.6 Prevalence of Ticks in Cattle Population

Prevalence of tick infestation was determined by the following formulae:

$$\mathrm{Prevalence}\;\left(\%\right)=\frac{\mathrm{no}.\;\mathrm{of}\;\mathrm{cattles}\;\mathrm{infested}\;\mathrm{with}\;\mathrm{ticks}}{\mathrm{total}\;\mathrm{no}.\;\mathrm{of}\;\mathrm{cattles}\;\mathrm{examined}}\times 100$$

Prevalence of different tick species:

$$\mathrm{Prevalence}\;\left(\%\right)=\frac{\mathrm{no}.\;\mathrm{of}\;a\;\mathrm{specific}\;\mathrm{specie}}{\mathrm{total}\;\mathrm{no}.\;\mathrm{of}\;\mathrm{ticks}\;\mathrm{examined}}\times 100$$

### 2.7 Epidemiological and Ecological Indices

To evaluate the association between potential risk factors (breed, age, and sex) and the likelihood of tick infestation, odds ratios (ORs) were calculated along with their 95% confidence intervals (CI). To determine whether tick species distribution varied significantly across cattle breeds, a chi‐square test of independence was applied. Tick burden was assessed based on the number of ticks collected per animal. Animals were categorized into three severity groups: mild (1–10 ticks), moderate (11–20 ticks), and severe (>20 ticks). To identify patterns of co‐infestation, each animal was examined for the presence of multiple tick species. Co‐occurrence of two or more species on the same host was recorded.

Species diversity among collected ticks was calculated using the Shannon–Weiner diversity index (H'), which accounts for both species richness and evenness. The formula used was

$$H^{\prime }=-\sum_{i=1}^{S}p_{i}\;ln\left(p_{i}\right)$$

where *p_i_* is the proportion of individuals belonging to the *i*th species, ln is the natural logarithm, and *S* is the total number of species.

To compare the dissimilarity in tick species composition between cattle breeds, the Bray–Curtis dissimilarity index was calculated using the formula:

$$BC_{ij}=1-\frac{2C_{ij}}{S_{i}+S_{j}}$$

where BC*_ij_* is the Bray–Curtis dissimilarity between breed *i* and breed *j*, *C_ij_* is the sum of the lesser counts for each species found in both breeds, and *S_i_*, *S_j_* is the total number of ticks in breeds *i* and *j*, respectively.

### 2.8 Molecular Detection of *T. annulata*

#### 2.8.1 DNA Extraction

Selected tick samples were triturated under sterile conditions, and genomic DNA was extracted using the GeneGet Genomic DNA Extraction Kit for Tissue (Cat. No. GG‐TD100, GeneGet Biotech, China), following the manufacturer's instructions. The quality and integrity of the extracted DNA were verified via 1.5% agarose gel electrophoresis before downstream applications.

#### 2.8.2 DNA Quantification

Quantification of the extracted DNA was performed using a Nanodrop spectrophotometer, measuring absorbance at 260/280 nm to assess concentration and purity.

### 2.9 PCR Amplification for *T. annulata* Detection

For the detection of *T. annulata*, PCR amplification was carried out targeting the Tams1‐1 gene, which serves as a species‐specific marker for the parasite. The primers used for this purpose were adopted from the protocol described. The forward primer sequence was 5′‐CCAGGACCACCCTCAAGTTC‐3′, and the reverse primer was 5′‐GCATCTAGTTCCTTGGCGGA‐3′. The amplification of this target region was expected to yield a product of 721 base pairs (bp) in size.

### 2.10 Agarose Gel Electrophoresis

The PCR products were resolved using a 1.5% agarose gel prepared in 1X TAE buffer. Ethidium bromide (0.5 µg/mL) was added to the gel for DNA visualization.

### 2.11 Gel Documentation and Analysis

**FIGURE 1:**
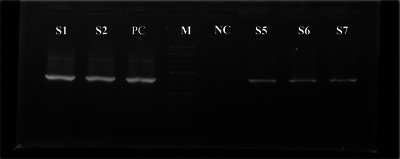
Positive result of PCR for *Theileria annulata* in ticks. M = DNA marker, S = Sample, NC = Negative control, PC = Positive control.

Following electrophoresis, the gels were visualized under ultraviolet light using a gel documentation system. The size of PCR products was compared to a 100 bp DNA ladder to confirm the amplification of the target gene (Figure 1).

### 2.12 Statistical Analysis

Data collected during the study were entered into Microsoft Excel and subsequently analyzed using IBM SPSS Statistics version 25.0 and PAST (Paleontological Statistics Software) for ecological indices. Descriptive statistics, including frequencies and percentages, were used to summarize tick prevalence, burden severity, co‐infestation patterns, and species composition.

The overall prevalence of tick infestation was calculated as the number of tick‐positive cattle divided by the total number examined (*n* = 322), expressed as a percentage. To assess the association between host‐related risk factors (breed, age, and sex) and the likelihood of tick infestation, binary logistic regression was performed. ORs with 95% CI were computed for each category compared to a reference group, and statistical significance was evaluated using Wald's test. Assumptions of logistic regression, including the absence of multicollinearity and linearity of log‐odds for continuous predictors, were checked prior to model interpretation. A *p*‐value < 0.05 was considered statistically significant.

Chi‐square tests of independence were conducted to determine the relationship between tick species distribution and cattle breed. This analysis aimed to identify species‐specific preferences for particular host breeds, and a *p*‐value threshold of < 0.05 indicated statistical significance. When expected cell frequencies were <5, Fisher's exact test was applied to ensure the validity of the results. The detection of *T. annulata* in ticks was analyzed using frequency‐based comparison between tick species and sexes. Fisher's exact test **or** Chi‐square test was employed where appropriate to test for differences in infection prevalence. Tick burden was classified into three severity levels (mild, moderate, and severe) based on tick count ranges per animal. The proportional distribution of animals in each category was calculated and presented descriptively. Patterns of tick co‐infestation were analyzed by identifying combinations of tick species on individual hosts. Frequencies and percentages were calculated to determine the most common co‐occurrence patterns.

Tick species diversity was assessed using the Shannon–Weiner diversity index (H′), which accounts for both species richness and evenness. To evaluate the compositional dissimilarity of tick species among different cattle breeds, the Bray–Curtis dissimilarity index was used. Rarefaction analysis was conducted to estimate and standardize species richness across cattle breeds with unequal sample sizes. Both observed and expected richness values were calculated using species accumulation data, allowing for meaningful comparison of biodiversity across breed groups. For multiple group comparisons (e.g., prevalence across different breeds and age categories), post‐hoc pairwise comparisons with Bonferroni correction were applied following significant Chi‐square tests to control for type I error. Data normality was checked using the Shapiro–Wilk test, and homogeneity of variances was evaluated using Levene's test. Where assumptions for parametric analysis were not met, data were either log‐transformed or analyzed using non‐parametric alternatives such as the Kruskal–Wallis test. All statistical tests were conducted at a 95% confidence level, and significance was declared at *p* < 0.05.

## 3 Results

**FIGURE 2:**
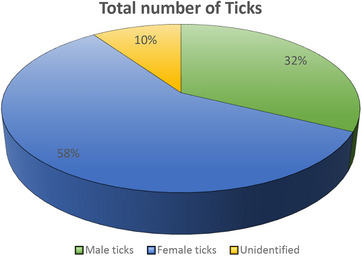
Ticks population collected from cattle in Peshawar, Pakistan.

A total of 322 cattle were examined for the presence of ticks. Out of 322 cattle examined, 114 were positive for tick infestation. The recorded tick prevalence was 35.4% (Figure 2).

The collected tick samples comprised both male and female specimens, along with a small proportion that could not be identified morphologically. As shown in Table 1, female ticks were more frequently encountered than males.

**TABLE 1:** Male and Female percentage of collected ticks in Peshawar, Pakistan.

Gender of ticks	Number	Percentage
Female	331	58.07%
Male	184	32.2%
Unidentified	55	9.6%
Total	570	

An analysis of various host‐related risk factors revealed notable differences in tick infestation prevalence among cattle breeds, age groups, and sexes, as summarized in Table 2. Among the breeds, exotic and crossbred cattle exhibited varying susceptibility levels. Calves displayed the highest infestation prevalence compared to older age groups, while sex‐based comparison did not yield statistically significant variation in infestation rates.

**TABLE 2:** Association of risk factors with ticks infestation in Peshawar, Pakistan.

Variables	Category	Positive	Prevalence(%)	*p*‐value
Breed	HF	39/81	48.14	0.007
	Jersy	25/69	36.23	
	Sahiwal	24/90	26.7	
	Cross Bred	26/82	31.7	
Age	Calf (1–12 months)	32/60	53.33	0.021
	12–24months	62/170	36.47	
	Adult	20/92	21.7	
Sex	Female	74/182	40.66	0.918
	Male	50/140	35.71	

**FIGURE 3:**
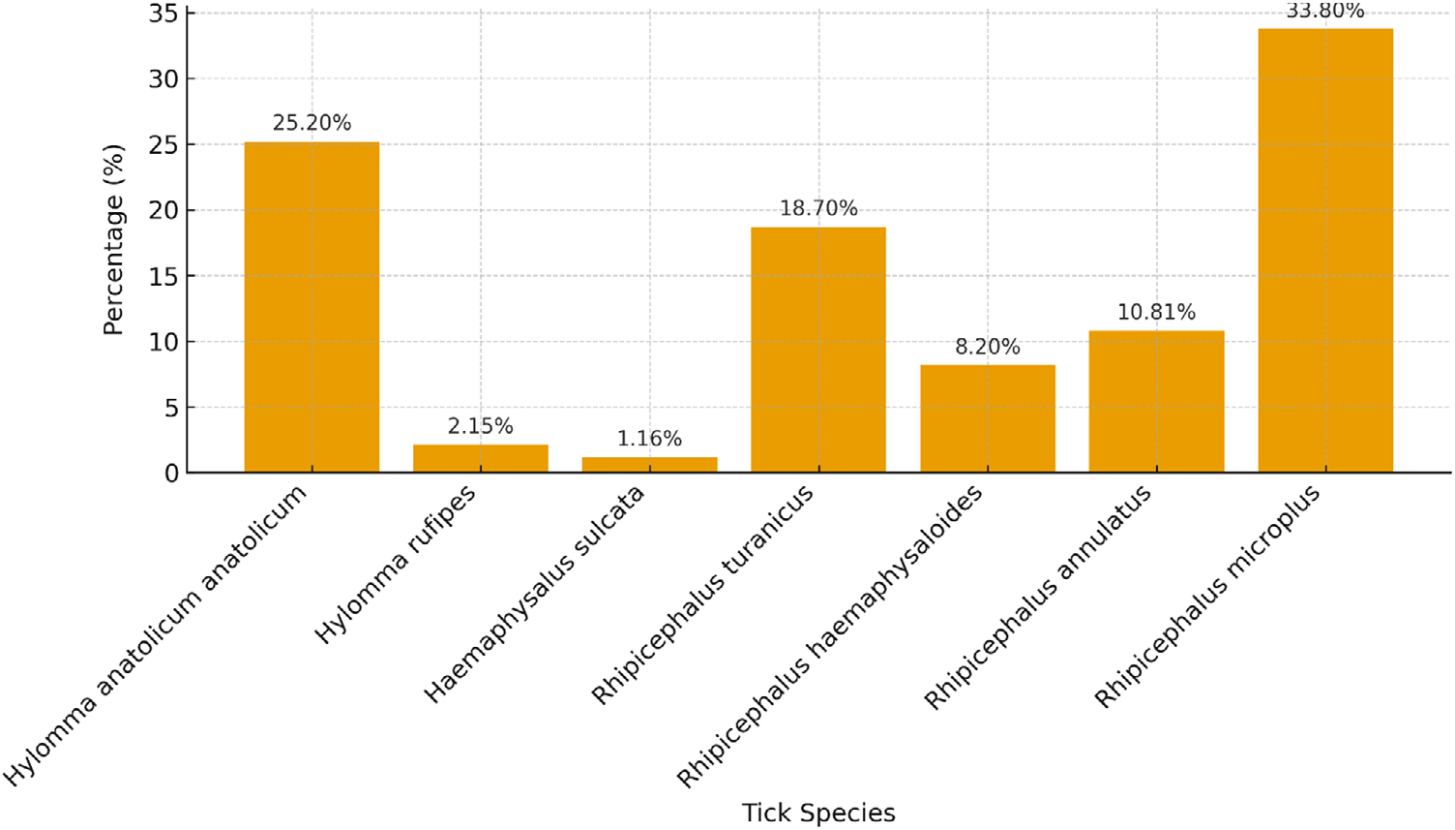
Percentage of each tick specie in total collected ticks in Peshawar, Pakistan.

The identification of tick species revealed a diverse tick fauna. According to Figure 3, *Rhipicephalus microplus* was the most predominant species, followed by *H. anatolicum anatolicum*, while other species were present at lower frequencies.

Risk assessment based on ORs and CI is presented in Table 3. Holstein Friesian and Jersey breeds showed higher odds of infestation in comparison to others, and age was also a significant risk factor, especially in younger cattle. No significant odds were observed when comparing between sexes.

**TABLE 3:** Odds ratios (OR) and confidence intervals for risk factors associated with tick infestation in Peshawar, Pakistan.

Variable	Category	OR (95% CI)	*p*‐value
Breed	Holstein Friesian vs. Others	2.25 (1.30–3.90)	0.01
Breed	Jersey vs. Others	1.60 (0.90–2.80)	0.08
Breed	Crossbred vs. Others	1.25 (0.70–2.20)	0.31
Age	Calf (1–12 months) vs. Adults	3.80 (1.90–7.60)	0.02
Age	12–24 months vs. Adults	2.10 (1.10–3.90)	0.04
Sex	Female vs. Male	1.22 (0.72–2.06)	0.91

Chi‐square analysis of tick species distribution by host breed, as depicted in Table 4, highlighted a strong association of *H. anatolicum anatolicum* with Holstein Friesian and *R. microplus* with Sahiwal cattle. *R. turanicus* showed no significant association with any specific breed.

**TABLE 4:** Chi‐square test for tick species distribution by breed in Peshawar, Pakistan.

Tick species	Highest prevalence in	Chi‐square value	*p*‐value
*H. anatolicum anatolicum*	Holstein Friesian Breed	6.15	0.01
*R. microplus*	Sahiwal Breed	4.59	0.03
*R. turanicus*	Crossbred	1.80	0.17
Other Species	—	—	—

Molecular screening for *T. annulata* infection, using ticks segregated by species and gender, is detailed in Table 5. Infection was more prevalent in *H. anatolicum anatolicum* females, while both sexes of *R. microplus* exhibited relatively low detection rates.

**TABLE 5:** Infection rate of *Theileria annulata* by tick gender in Peshawar, Pakistan.

Tick species	Gender	Number tested	Positive (%)	*p*‐value
*H. anatolicum anatolicum*	Female	90	25.5	0.01
*H. anatolicum anatolicum*	Male	40	15.0	0.32
*R. microplus*	Female	40	7.5	0.65
*R. microplus*	Male	16	6.2	0.98

Tick burden among infested animals varied in severity, as outlined in Table 6. The majority of animals experienced moderate infestation levels, with fewer animals presenting either mild or severe infestations.

**TABLE 6:** Tick burden severity categories in cattle in Peshawar, Pakistan.

Severity level	Tick count range	Number of animals	Percentage (%)
Mild	1–10	90	35.2
Moderate	11–20	110	43.0
Severe	>20	55	21.8
Total	—	255	100

Patterns of co‐infestation among tick species are documented in Table 7. The most frequent combination involved *H. anatolicum* and *R. microplus*, whereas a large proportion of animals hosted only a single tick species.

**TABLE 7:** Co‐infestation patterns of tick species in Peshawar, Pakistan.

Combination of tick species	Number of animals	Percentage (%)
*H. anatolicum* + *R. microplus*	38	14.9
*R. turanicus* + *R. microplus*	20	7.8
*H. anatolicum* + *R. turanicus*	12	4.7
No co‐infestation	185	72.5

Diversity of the tick population was assessed using the Shannon–Weiner Index, presented in Table 8. The overall species diversity was moderate, with contributions varying by species. *R. microplus* and *H. anatolicum* contributed the most to the overall diversity index.

**TABLE 8:** Shannon–Weiner Diversity Index (H') for tick species collected in Peshawar, Pakistan.

Tick species	Number (n_i_)	Proportion (p_i_)	pi × ln (pi)
*H. anatolicum anatolicum*	130	0.25	−0.34
*H. rufipes*	11	0.02	−0.08
*Haemaphysalis sulcata*	6	0.01	−0.05
*R. turanicus*	96	0.18	−0.31
*R. haemaphysaloides*	42	0.08	−0.18
*R. annulatus*	56	0.10	−0.24
*R. microplus*	174	0.33	−0.36
Total	515	1.00	−1.59

Comparative analysis of species composition across breeds using Bray–Curtis dissimilarity indices is given in Table 9. The greatest dissimilarity in tick species distribution was noted between Holstein Friesian and Sahiwal breeds, while crossbred cattle shared more similarities with other breeds.

**TABLE 9:** Bray–Curtis dissimilarity of tick species composition between cattle breeds in Peshawar, Pakistan.

Breed comparison	Bray–Curtis Dissimilarity Index
HF vs. Jersey	0.42
HF vs. Sahiwal	0.51
HF vs. Crossbred	0.37
Jersey vs. Sahiwal	0.44
Jersey vs. Crossbred	0.33
Sahiwal vs. Crossbred	0.29

Rarefaction analysis, as summarized in Table 10, indicated that Holstein Friesian cattle exhibited the highest observed and standardized species richness, while crossbred cattle had the lowest values, suggesting breed‐based differences in exposure or susceptibility to multiple tick species.

**TABLE 10:** Rarefaction analysis for tick species richness by breed in Peshawar, Pakistan.

Breed	Individuals sampled	Observed species richness	Expected species richness (standardized)
HF	81	6	5.8
Jersey	69	5	5.0
Sahiwal	90	5	5.3
Crossbred	82	4	4.1

## 4 Discussion

The observed prevalence of tick infestation in cattle from District Peshawar aligns with findings reported in similar agro‐ecological zones of Pakistan (Ghafar et al. 2020), suggesting that regional environmental and management conditions continue to support moderate tick burdens. Warm temperatures, communal grazing, and limited tick control measures likely sustain endemic tick populations. This reinforces the need for context‐specific interventions in such regions.

The predominance of female ticks over males is consistent with earlier reports (Rehman et al. 2017) and reflects biological differences in feeding behavior and reproduction. Female ticks’ longer attachment and higher blood meal requirements increase their visibility and likelihood of detection. Moreover, their ability to release aggregation pheromones enhances their capacity to remain on hosts (Estrada‐Peña et al. 2004; Sonenshine 2004), underlining their critical role in tick population dynamics and pathogen transmission cycles.

The study further confirms the heightened vulnerability of exotic and crossbred cattle compared to the indigenous Sahiwal. This differential susceptibility likely stems from breed‐specific physiological and immunological traits, such as thinner skin and weaker innate defenses in exotic breeds (Tabor et al. 2017). These observations emphasize the importance of breed selection and targeted tick management, particularly when integrating non‐native breeds into endemic areas. The apparent resistance of Sahiwal cattle also supports their continued use in tick‐prone regions, as echoed in previous findings (A. Iqbal et al. 2013).

Calves’ higher infestation rates, commonly attributed to immature immune systems and softer skin, are in line with established literature (Robbertse et al. 2017; Tabor et al. 2017). This highlights the need for early‐life tick control strategies to prevent not only infestation but also early exposure to vector‐borne pathogens. Conversely, the lack of a significant difference between male and female cattle indicates that sex may not be a critical factor under similar husbandry and exposure conditions, corroborating findings by Durrani et al. (2008).

In terms of species composition, *R. microplus* emerged as the dominant tick, followed by *H. anatolicum*. The success of *R. microplus* may be attributed to its one‐host lifecycle and high reproductive efficiency, traits that facilitate rapid population expansion. On the other hand, *H. anatolicum* is a well‐documented vector of *T. annulata* and is favored by environmental conditions in semi‐arid zones (S. Ali et al. 2024). Its immunomodulatory salivary components (A. Ali et al. 2022) further enhance its vectorial capacity.

The identification of multiple tick species co‐infesting the same host highlights ecological overlaps and potential for co‐transmission of pathogens—a growing concern in livestock health. However, the majority of animals hosted only a single tick species, possibly indicating competitive exclusion or species‐specific host preferences (Estrada‐Peña et al. 2004). These dynamics complicate control efforts, as co‐infestation may increase pathogen diversity and virulence.

Host‐tick specificity was further evidenced by significant associations between certain tick species and cattle breeds. For instance, *H. anatolicum* was more prevalent in Holstein Friesian cattle, whereas *R. microplus* was frequently found on Sahiwal cattle. This specificity may reflect a complex interplay of host skin texture, behavior, and chemical cues (Estrada‐Peña et al. 2004) and calls for deeper investigation into host‐attractant mechanisms to inform breed‐specific interventions.

The detection of *T. annulata* predominantly in female *H. anatolicum* reinforces the role of this species as a primary vector, particularly females, due to their prolonged feeding periods. The lower infection rates in *R. microplus* align with its known association with *Babesia* spp. rather than *Theileria* spp. (Marques et al. 2020), suggesting limited competence for *T. annulata* in this region. These findings are critical in refining vector surveillance and targeting the most epidemiologically relevant tick species.

Tick burden assessment revealed a predominance of moderate infestations, which is typical for areas where tick pressure is continuous but subclinical. Similar patterns have been reported in related ecological contexts, indicating partial host tolerance or seasonal fluctuation in tick populations. The moderate severity suggests a manageable risk but still underscores the importance of consistent control practices.

Co‐infestation patterns, especially the frequent pairing of *H. anatolicum* and *R. microplus*, reflect the ecological compatibility and shared host preferences of these ticks (Zeb et al. 2019). Their coexistence elevates the risk of simultaneous transmission of multiple pathogens, potentially complicating diagnosis and treatment. The apparent absence of complex multi‐species infestations may suggest interspecific competition or niche partitioning.

Ecological diversity metrics, such as the Shannon–Weiner index, indicated a moderately diverse tick community, dominated by the two aforementioned species. This limited diversity may reduce the complexity of vector control but still poses a substantial risk due to the high vector competence of the dominant species (Hussain et al. 2024).

Finally, the Bray–Curtis dissimilarity analysis demonstrated clear variation in tick species composition across cattle breeds, further supporting the influence of breed‐related factors on tick ecology. The higher species richness observed in Holstein Friesian cattle could be attributed to their heightened attractiveness and susceptibility to ticks. In contrast, crossbreds showed lower richness, possibly due to hybrid vigor or intermediate resistance. These findings support the development of breed‐tailored control strategies and reinforce the importance of host genetic factors in TBD epidemiology.

## 5 Conclusion

This study recorded a 35.4% tick infestation rate in cattle, with higher susceptibility in exotic breeds and calves in Peshawar District. *R. microplus* and *H. anatolicum anatolicum* were the dominant species, the latter showing a stronger association with *T. annulata* infection. These findings highlight the need for targeted tick control measures, especially in high‐risk breeds and younger animals.

## Author Contributions

**Murad Ali Khan**: conceptualization, methodology, validation, visualization, funding acquisition, software, formal analysis. **Zohaib Ali**: investigation, methodology, software. **Abdur Rehman**: project administration. **Shaban Naz** and **Maro Ragni**: formal analysis, data curation, supervision. **Murad Ali Khan**: supervision. **Solomon Tesfay**: investigation. **Rifat Ullah Khan**: data curation, software, methodology. **Shabana Naz**: writing–original draft, writing–review and editing.

## Ethics Statement

All the experimental procedures adopted in this study were pre‐approved by the animal welfare and care committee on the use of experimental animals at the University of Agriculture Peshawar, Pakistan (No. FAHVS/33/2022)

## Consent

The authors have nothing to report.

## Conflicts of Interest

The authors declared there is no conflict of interest

## Peer Review

The peer review history for this article is available at https://www.webofscience.com/api/gateway/wos/peer‐review/10.1002/vms3.70642.

## Data Availability

The authors have nothing to report.
